# Supplementary material for: Trait Energy and Fatigue Modify the Effects of Caffeine on Mood, Cognitive and Fine-Motor Task Performance: A Post-Hoc Study
Source: Nutrients. 2021 Jan 28;13(2):412. doi: 10.3390/nu13020412 (PMC7912474; doi:10.3390/nu13020412)
Supplement: Supplementary file 1 [file nutrients-13-00412-s001.pdf]

FOR SUPPLEMENTAL MATERIALS

Trait and caffeine influence on moods, cognitive tasks and fine motor tasks (all findings with significant differences are in bold)

Factor	Measure	Beta	2.5%	97.5%	t stat	p value
**TPE**	**Vigor**	**-3.234**	**-5.686**	**-0.783**	**-2.586**	**0.014**
Caffeine	Vigor	0.763	-0.221	1.747	1.520	0.132
TPE x Caffeine	Vigor	-0.536	-2.161	1.089	-0.646	0.520
TPF	Vigor	0.400	-2.266	3.066	0.294	0.771
**Caffeine**	**Vigor**	**1.300**	**0.211**	**2.389**	**2.341**	**0.022**
TPF x Caffeine	Vigor	-1.467	-3.006	0.073	-1.867	0.065
TME	Vigor	-2.545	-5.446	0.355	-1.720	0.095
Caffeine	Vigor	0.432	-0.483	1.347	0.925	0.357
TME x Caffeine	Vigor	0.506	-1.266	2.277	0.559	0.577
TMF	Vigor	0.451	-2.216	3.117	0.331	0.742
**Caffeine**	**Vigor**	**1.437**	**0.396**	**2.479**	**2.706**	**0.008**
**TMF x Caffeine**	**Vigor**	**-1.866**	**-3.390**	**-0.342**	**-2.400**	**0.019**
TPE	Fatigue	1.730	0.0550	3.404	2.025	0.050
Caffeine	Fatigue	-0.105	-0.879	0.669	-0.267	0.790
TPE x Caffeine	Fatigue	-0.031	-1.310	1.247	-0.048	0.962
TPF	Fatigue	0.633	-1.049	2.316	0.738	0.465
Caffeine	Fatigue	-0.500	-1.364	0.364	-1.134	0.260
TPF x Caffeine	Fatigue	0.767	-0.455	1.988	1.230	0.222
TME	Fatigue	0.409	-1.537	2.355	0.412	0.683
Caffeine	Fatigue	-0.114	-0.833	0.606	-0.310	0.758
TME x Caffeine	Fatigue	-0.011	-1.405	1.382	-0.016	0.987
TMF	Fatigue	0.228	-1.469	1.924	0.263	0.794
Caffeine	Fatigue	-0.688	-1.513	0.138	-1.633	0.106
TMF x Caffeine	Fatigue	1.223	0.015	2.431	1.984	0.050
TPE	Tension	0.419	-0.182	1.019	1.367	0.178
Caffeine	Tension	0.263	-0.080	0.607	1.502	0.137
TPE x Caffeine	Tension	-0.081	-0.648	0.486	-0.281	0.779
TPF	Tension	0.367	-0.423	0.356	1.222	0.229
**Caffeine**	**Tension**	**0.633**	**0.265**	**1.001**	**3.372**	**0.001**
**TPF x Caffeine**	**Tension**	**-0.800**	**-1.321**	**-0.279**	**-3.012**	**0.003**
TME	Tension	0.199	-0.363	0.699	0.583	0.563
Caffeine	Tension	0.250	-0.069	0.569	1.535	0.128
TME x Caffeine	Tension	-0.062	-0.681	0.556	-0.198	0.843
TMF	Tension	0.357	-0.194	0.605	1.187	0.242
Caffeine	Tension	0.375	0.003	0.747	1.977	0.051
TMF x Caffeine	Tension	-0.304	-0.848	0.241	-1.093	0.277
TPE	Anger	0.115	-0.357	0.587	0.477	0.635
Caffeine	Anger	0.211	-0.128	0.549	1.220	0.226
TPE x Caffeine	Anger	0.471	-0.087	1.030	1.654	0.102
TPF	Anger	0.067	-0.407	0.541	0.276	0.784
**Caffeine**	**Anger**	**0.400**	**0.013**	**0.787**	**2.028**	**0.046**
TPF x Caffeine	Anger	-0.033	-0.58	0.513	-0.120	0.905
TME	Anger	-0.187	-0.721	0.346	-0.689	0.493
**Caffeine**	**Anger**	**0.364**	**0.045**	**0.683**	**2.234**	**0.028**
TME x Caffeine	Anger	0.074	-0.544	0.692	0.234	0.815
TMF	Anger	0.161	-0.310	0.632	0.669	0.506

Caffeine	Anger	0.375	0.001	0.749	1.964	0.053
TMF x Caffeine	Anger	0.018	-0.530	0.566	0.064	0.949
TPE	Depression	0.244	-0.241	0.729	0.986	0.331
Caffeine	Depression	-0.053	-0.241	0.136	-0.548	0.585
TPE x Caffeine	Depression	0.053	-0.258	0.364	0.332	0.741
TPF	Depression	0.400	-0.064	0.864	1.691	0.100
Caffeine	Depression	0.067	-0.143	0.277	0.622	0.536
TPF x Caffeine	Depression	-0.200	-0.497	0.097	-1.319	0.190
TME	Depression	0.358	-0.158	0.874	1.360	0.183
Caffeine	Depression	-0.068	-0.243	0.106	-0.765	0.446
TME x Caffeine	Depression	0.131	-0.207	0.469	0.757	0.451
TMF	Depression	0.442	-0.024	0.908	1.858	0.072
Caffeine	Depression	0.125	-0.075	0.325	1.227	0.223
**TMF x Caffeine**	**Depression**	**-0.339**	**-0.632**	**-0.047**	**-2.276**	**0.025**
**TPE**	**Confusion**	**0.830**	**0.033**	**1.628**	**2.040**	**0.047**
Caffeine	Confusion	-0.053	-0.557	0.452	-0.205	0.838
TPE x Caffeine	Confusion	0.507	-0.326	1.340	1.194	0.236
TPF	Confusion	0.500	-0.344	1.344	1.160	0.252
Caffeine	Confusion	0.167	-0.405	0.739	0.571	0.569
TPF x Caffeine	Confusion	-0.067	-0.876	0.742	-0.162	0.872
TME	Confusion	0.500	-0.460	1.460	1.021	0.313
Caffeine	Confusion	0.159	-0.313	0.631	0.660	0.511
TME x Caffeine	Confusion	-0.097	-1.011	0.818	-0.207	0.836
TMF	Confusion	0.420	-0.432	1.271	0.966	0.339
Caffeine	Confusion	0.156	-0.398	0.710	0.553	0.582
TMF x Caffeine	Confusion	-0.049	-0.860	0.762	-0.119	0.906
**TPE**	**Motivation**	**-1.469**	**-2.827**	**-0.111**	**-2.121**	**0.040**
Caffeine	Motivation	0.053	-0.627	0.732	0.152	0.880
TPE x Caffeine	Motivation	0.402	-0.721	1.525	0.702	0.485
TPF	Motivation	0.067	-1.314	1.448	0.095	0.925
Caffeine	Motivation	0.500	-0.262	1.262	1.286	0.202
TPF x Caffeine	Motivation	-0.600	-1.678	0.478	-1.091	0.278
TME	Motivation	-0.517	-2.076	1.042	-0.65	0.520
Caffeine	Motivation	0.136	-0.497	0.769	0.422	0.674
TME x Caffeine	Motivation	0.239	-0.987	1.465	0.382	0.704
TMF	Motivation	0.478	-0.902	1.858	0.679	0.502
**Caffeine**	**Motivation**	**0.812**	**0.094**	**1.531**	**2.215**	**0.029**
**TMF x Caffeine**	**Motivation**	**-1.312**	**-2.365**	**-0.260**	**-2.444**	**0.017**
TPE	Physical Energy	1.842	-1.057	4.742	1.245	0.218
Caffeine	Physical Energy	0.526	-1.410	2.463	0.533	0.596
TPE x Caffeine	Physical Energy	-2.072	-5.270	1.127	-1.270	0.208
TPF	Physical Energy	1.200	-1.607	4.007	0.838	0.406
Caffeine	Physical Energy	0.900	-1.274	3.074	0.811	0.419
TPF x Caffeine	Physical Energy	-2.267	-5.341	0.808	-1.445	0.152
TME	Physical Energy	-0.284	-3.450	2.881	-0.176	0.861
Caffeine	Physical Energy	0.091	-1.721	1.902	0.098	0.922
TME x Caffeine	Physical Energy	-1.216	-4.724	2.292	-0.679	0.499
TMF	Physical Energy	0.714	-2.105	3.534	0.497	0.621
Caffeine	Physical Energy	0.094	-2.034	2.221	0.086	0.931
TMF x Caffeine	Physical Energy	-0.701	-3.815	2.413	-0.441	0.660
**TPE**	**Physical Fatigue**	**-4.926**	**-8.137**	**-1.715**	**-3.007**	**0.004**
Caffeine	Physical Fatigue	0.974	-1.236	3.183	0.864	0.390

TPE x Caffeine	Physical Fatigue	2.799	-0.850	6.448	1.503	0.136
TPF	Physical Fatigue	0.167	-3.105	3.438	0.100	0.921
Caffeine	Physical Fatigue	0.400	-2.074	2.874	0.317	0.752
TPF x Caffeine	Physical Fatigue	3.200	-0.298	6.698	1.793	0.076
TME	Physical Fatigue	-2.875	-6.623	0.873	-1.503	0.139
Caffeine	Physical Fatigue	0.568	-1.429	2.565	0.558	0.579
**TME x Caffeine**	**Physical Fatigue**	**5.369**	**1.502**	**9.237**	**2.721**	**0.008**
TMF	Physical Fatigue	0.701	-2.604	4.005	0.416	0.679
Caffeine	Physical Fatigue	1.188	-1.238	3.613	0.959	0.340
TMF x Caffeine	Physical Fatigue	1.741	-1.810	5.292	0.961	0.339
TPE	Mental Energy	1.108	-1.862	4.077	0.731	0.468
Caffeine	Mental Energy	0.474	-1.445	2.393	0.484	0.630
TPE x Caffeine	Mental Energy	-1.474	-4.643	1.695	-0.911	0.365
TPF	Mental Energy	0.100	-2.740	2.940	0.069	0.945
Caffeine	Mental Energy	0.967	-1.182	3.115	0.882	0.380
TPF x Caffeine	Mental Energy	-2.067	-5.105	0.972	-1.333	0.186
TME	Mental Energy	-0.500	-3.718	2.718	-0.304	0.762
Caffeine	Mental Energy	0.227	-1.561	2.015	0.249	0.804
TME x Caffeine	Mental Energy	-1.102	-4.564	2.360	-0.624	0.534
TMF	Mental Energy	-0.487	-3.340	2.367	-0.334	0.740
Caffeine	Mental Energy	0.344	-1.753	2.441	0.321	0.749
TMF x Caffeine	Mental Energy	-0.879	-3.950	2.191	-0.561	0.576
**TPE**	**Mental Fatigue**	**-4.483**	**-7.678**	**-1.289**	**-2.751**	**0.008**
Caffeine	Mental Fatigue	1.026	-1.206	3.258	0.901	0.370
TPE x Caffeine	Mental Fatigue	1.974	-1.712	5.660	1.049	0.297
TPF	Mental Fatigue	0.333	-2.921	3.587	0.201	0.842
Caffeine	Mental Fatigue	0.367	-2.128	2.861	0.288	0.774
TPF x Caffeine	Mental Fatigue	2.767	-0.761	6.294	1.537	0.128
TME	Mental Fatigue	-2.562	-6.286	1.161	-1.349	0.183
Caffeine	Mental Fatigue	0.364	-1.647	2.374	0.355	0.724
**TME x Caffeine**	**Mental Fatigue**	**5.199**	**1.306**	**9.092**	**2.617**	**0.010**
TMF	Mental Fatigue	0.746	-2.543	4.034	0.444	0.659
Caffeine	Mental Fatigue	1.094	-1.345	3.533	0.879	0.382
TMF x Caffeine	Mental Fatigue	1.406	-2.164	4.977	0.772	0.442
TPE	Sub3total	4.909	-1.770	11.588	1.441	0.159
Caffeine	Sub3total	-0.289	-2.626	2.047	-0.243	0.809
TPE x Caffeine	Sub3total	0.153	-3.706	4.012	0.078	0.938
TPF	Sub3total	-0.333	-6.960	6.294	-0.099	0.922
Caffeine	Sub3total	-2.433	-4.982	0.115	-1.872	0.065
**TPF x Caffeine**	**Sub3total**	**4.400**	**0.796**	**8.004**	**2.393**	**0.019**
**TME**	**Sub3total**	**9.222**	**2.045**	**16.398**	**2.519**	**0.017**
Caffeine	Sub3total	1.432	-0.633	3.496	1.359	0.177
**TME x Caffeine**	**Sub3total**	**-6.244**	**-10.242**	**-2.247**	**-3.062**	**0.003**
TMF	Sub3total	-1.165	-7.804	5.474	-0.344	0.733
**Caffeine**	**Sub3total**	**-2.875**	**-5.290**	**-0.46**	**-2.334**	**0.022**
**TMF x Caffeine**	**Sub3total**	**5.661**	**2.126**	**9.196**	**3.139**	**0.002**
TPE	Sub7total	2.208	-3.389	7.806	0.773	0.445
**Caffeine**	**Sub7total**	**2.632**	**0.518**	**4.746**	**2.440**	**0.017**
TPE x Caffeine	Sub7total	-0.859	-4.350	2.632	-0.482	0.631
TPF	Sub7total	0.767	-4.629	6.162	0.279	0.782
Caffeine	Sub7total	1.433	-0.935	3.801	1.186	0.239
TPF x Caffeine	Sub7total	1.767	-1.582	5.116	1.034	0.304

**TME**	**Sub7total**	**6.858**	**1.106**	**12.61**	**2.337**	**0.025**
**Caffeine**	**Sub7total**	**2.886**	**0.933**	**4.840**	**2.896**	**0.005**
TME x Caffeine	Sub7total	-2.136	-5.920	1.647	-1.107	0.271
TMF	Sub7total	-1.937	-7.354	3.479	-0.701	0.488
Caffeine	Sub7total	0.000	-2.195	2.195	<0.001	>0.999
**TMF x Caffeine**	**Sub7total**	**4.964**	**1.751**	**8.178**	**3.028**	**0.003**
TPE	DH	2.024	-10.234	14.282	0.324	0.748
Caffeine	DH	-1.368	-5.254	2.517	-0.690	0.492
TPE x Caffeine	DH	-0.132	-6.548	6.285	-0.040	0.968
TPF	DH	6.733	-5.036	18.503	1.121	0.271
Caffeine	DH	3.300	-0.845	7.445	1.560	0.122
**TPF x Caffeine**	**DH**	**-9.433**	**-15.295**	**-3.572**	**-3.154**	**0.002**
TME	DH	2.097	-11.121	15.314	0.311	0.758
Caffeine	DH	-3.136	-6.679	0.407	-1.735	0.086
TME x Caffeine	DH	6.449	-0.412	13.31	1.842	0.069
TMF	DH	3.000	-8.813	14.813	0.498	0.622
Caffeine	DH	2.719	-1.322	6.759	1.319	0.191
**TMF x Caffeine**	**DH**	**-8.862**	**-14.777**	**-2.947**	**-2.936**	**0.004**
TPE	NDH	6.612	-5.652	18.877	1.057	0.299
Caffeine	NDH	0.237	-3.435	3.909	0.126	0.900
TPE x Caffeine	NDH	-5.600	-11.665	0.464	-1.81	0.074
TPF	NDH	1.267	-10.571	13.105	0.210	0.835
Caffeine	NDH	2.200	-1.838	6.238	1.068	0.289
**TPF x Caffeine**	**NDH**	**-8.033**	**-13.744**	**-2.322**	**-2.757**	**0.007**
TME	NDH	4.409	-8.908	17.727	0.649	0.521
Caffeine	NDH	-2.341	-5.810	1.128	-1.323	0.189
TME x Caffeine	NDH	1.966	-4.752	8.684	0.574	0.568
TMF	NDH	-2.844	-14.481	8.793	-0.479	0.635
Caffeine	NDH	1.906	-2.007	5.819	0.955	0.342
**TMF x Caffeine**	**NDH**	**-7.978**	**-13.706**	**-2.250**	**-2.730**	**0.008**

TPE= Trait Physical Energy, TPF= Trait Physical Fatigue, TME= Trait Mental Energy, TMF= Trait Mental Fatigue, DH= Dominant Hand, NDH= Non-dominant hand
